# Retention of Activity by Antibodies Immobilized on Gold Nanoparticles of Different Sizes: Fluorometric Method of Determination and Comparative Evaluation

**DOI:** 10.3390/nano11113117

**Published:** 2021-11-18

**Authors:** Dmitriy V. Sotnikov, Nadezhda A. Byzova, Anatoly V. Zherdev, Boris B. Dzantiev

**Affiliations:** Research Center of Biotechnology of the Russian Academy of Sciences, A.N. Bach Institute of Biochemistry, 119071 Moscow, Russia; sotnikov-d-i@mail.ru (D.V.S.); nbyzova@inbi.ras.ru (N.A.B.); zherdev@inbi.ras.ru (A.V.Z.)

**Keywords:** immunoglobulins, gold nanoparticles, bioconjugation, tryptophan, fluorescence, surface coverage

## Abstract

Antibody–nanoparticle conjugates are widely used analytical reagents. An informative parameter reflecting the conjugates’ properties is the number of antibodies per nanoparticle that retain their antigen-binding ability. Estimation of this parameter is characterized by a lack of simple, reproducible methods. The proposed method is based on the registration of fluorescence of tryptophan residues contained in proteins and combines sequential measurements of first the immobilized antibody number and then the bound protein antigen number. Requirements for the measurement procedure have been determined to ensure reliable and accurate results. Using the developed technique, preparations of spherical gold nanoparticles obtained by the most common method of citrate reduction of gold salts (the Turkevich–Frens method) and varying in average diameter from 15 to 55 nm have been characterized. It was shown that the number of antibodies (immunoglobulins G) bound by one nanoparticle ranged from 30 to 194 during adsorptive unoriented monolayer immobilization. C-reactive protein was considered as the model antigen. The percentage of antibody valences that retained their antigen-binding properties in the conjugate increased from 17 to 34% with an increase in the diameter of gold nanoparticles. The proposed method and the results of the study provide tools to assess the capabilities of the preparations of gold nanoparticles and their conjugates as well as the expediency of seeking the best techniques for various practical purposes.

## 1. Introduction

Antibody–marker complexes are effective reagents for bioanalytical purposes. They combine high-affinity and selective antibody interaction with the target analyte and highly sensitive detection of the formed complex, provided by the marker’s properties [1]. Among the various markers, recently nanoparticles have been actively studied. Their use makes it possible to implement various detection methods and register immune complexes at extremely low concentrations [2,3,4]. The significant variety of nanoparticles differing in size, shape, chemical composition, and other factors as well as the variety of methods for their complexation with antibodies determine the significant variability of the resulting conjugates. The problems of antibody inactivation during immobilization with the subsequent deterioration of immunosensoric systems’ analytical characteristics have been noted in many studies and have prompted various approaches for indirect oriented antibody immobilization [5,6,7,8,9]. However, such actions involve the use of additional reagents and methodological complications. Therefore, many variants of direct immobilization on nanoparticles continue to be widely used, and assessments of the resulting preparations’ functional properties are in high demand. The most informative parameters for such characteristics are the composition of antibody–nanoparticle conjugates and the content of both inactive antibodies (that have lost their antigen-binding properties after conjugation) and reactive ones. These parameters allow for the assessment of the possibilities of analytical use for the conjugates obtained and the comparison of new and commonly used reactants.

The number of molecules immobilized on the surface of a nanoparticle has been measured in several works [10,11,12,13,14,15,16,17,18,19,20,21]. To complete this task, a variety of methods have been proposed: spectroscopic (absorption [11], emission [12], fluorescent [13,14], etc.), mass spectrometry [15], light scattering (Raman [16], dynamic [17], etc.), analytical separation (chromatography [18], electrophoresis [19], ultracentrifugation [20], field-flow fractioning [21]), and others. The retention degree of the antibodies’ reactivity in conjugates is characterized to a much lesser extent. Most researchers limit the testing of conjugates by comparing the concentration dependences of their binding with antigens, including their direct use in a specific analytical system [22,23,24,25,26]. Such a comparison solves the particular problem of the choice for the given analytical system but is not informative to assess the conjugates’ intrinsic properties, which are manifested differently at different modes of interaction with the antigen. Direct measurement of the number of antibodies on the surface of a nanoparticle that has retained its antigen-binding ability has been carried out in only a few works [27,28,29,30], which are discussed in detail below.

Three main criteria should be considered when evaluating the capabilities of different conjugate characterization methods:

- Methodological simplicity (because each manipulation stage reduces the accuracy of the final measurements)
- The absence of chemical modifications or harsh treatments that may affect the resulting preparations’ interaction parameters and properties
- Productivity, providing simultaneous testing of many preparations and reliable results after the statistical processing of replicates

In this regard, methodological solutions based on the assessment of the concentrations of (i) antibodies or antigens in the initial solution added for immobilization on native nanoparticles or binding to the conjugated antibodies and (ii) unbound antibodies (antigens) separated from the reaction product seem promising. Such an assessment of concentrations can be carried out by the intrinsic optical properties of antibodies or antigens or by their specific immunochemical detection. Earlier in [13], the characterization of the binding of antibodies to gold nanoparticles (GNPs) was carried out through the registration of fluorescence of tryptophan amino acid residues. In [28], the binding of the antigen peroxidase by antibodies immobilized on the surface of a nanoparticle was assessed using enzyme immunoassay.

However, the earlier studies based on the detection of tryptophan fluorescence to characterize proteins binding with nanoparticles [31,32] were limited by the formation of the first layer at the nanoparticle surface and considered only quantity of bound molecules without assessment of their functional activity.

In this work more complicated system of interacting compounds (nanoparticle + antibody + antigen) is considered. We propose an integral technique in which both the binding of antibodies to nanoparticles and the subsequent interaction of a protein antigen with immobilized antibodies are characterized by the same approach: registration of tryptophan fluorescence. Tryptophan is the most intensely fluorescent amino acid, with maximum absorption at 280 nm and maximum emission at 340–360 nm [33]. Tryptophan is contained in almost all proteins and is used to assess their concentrations in various biochemical studies [34]. Therefore, tryptophan fluorescence allows for the analysis of a wide range of conjugates without the need for additional reactants.

This study combines the characterization of the proposed method and its assessment of the number of reactive antibodies adsorbed on the surface of GNPs, with varying particle sizes and antibody surface densities. The Turkevich–Frens method, the most widespread method for obtaining spherical GNPs by the reduction of gold salt in a solution using citrate ions [35,36,37,38], was chosen for this study. The characterized GNP panel included 6 preparations of different diameters, from 15 to 55 nm. Antibodies were conjugated with GNPs under conditions that ensured surface saturation with the formation of a monolayer and by considering lower antibody loads. The antigen characterized was a C-reactive protein (CRP), a diagnostically significant marker of inflammatory processes [39,40].

## 2. Materials and Methods

### 2.1. Materials

Monoclonal antibodies (IgG) against CRP, clone C2cc, and native CRP by HyTest (Moscow, Russia) were used. Gold (III) chloride hydrate (HAuCl_4_), Tris, and sodium citrate were purchased from Sigma-Aldrich (St. Louis, MO, USA). Monofunctional poly(ethylene glycol) with a reactive free thiol (PEG-SH; MW 5 k) was purchased from Creative PEGWorks (Durham, NC, USA). All salts and acids were purchased from Khimmed (Moscow, Russia). Deionized water produced by Milli-Q (Millipore; Burlington, MA, USA) was used to prepare buffers.

### 2.2. Preparation and Characterization of GNPs

Six preparations of spherical GNPs were synthesized according to [36], taking into account the choice of reagent concentrations considered in our previous work [41]. A 1% HAuCl_4_ solution was added to deionized water and brought to a boil, and a 1% sodium citrate solution was added with stirring. Upon receipt of 6 preparations, the sodium citrate solution volumes were 3.0, 2.0, 1.75, 1.5, 1.0, and 0.75 mL; the HAuCl_4_ solution volume was constant and equal to 1.0 mL; and the water volume was chosen to reach 100 mL of the final reaction mixture. The mixture was boiled for 25 min, then cooled and stored at 4–6 °C. The preparations obtained were designated as GNP1–GNP6.

### 2.3. Determination of the Dimensional Characteristics of GNPs by Transmission Electron Microscopy (TEM)

GNP preparations were applied to nets (300 mesh) covered by a polyvinyl formal film dissolved in chloroform. The images were obtained with a JEM CX-100 electron microscope (Jeol; Tokyo, Japan) at an accelerating voltage of 80 kV and a magnification of 3,300,000. The digital photographs were analyzed using Image Tool software (University of Texas Health Science Center, San Antonio, TX, USA).

### 2.4. Spectral Characteristics of GNPs

The absorption spectra of the obtained GNP preparations were in the wavelength range of 400–700 nm and were recorded on a Libra S80 spectrophotometer (Biochrom; Cambridge, UK).

### 2.5. Obtaining GNP Conjugates with Antibodies against CRPs of Different Compositions

Using the synthesized GNP1–GNP6 preparations, conjugates with IgG of clone C2cc against CRP were obtained by adsorption immobilization. Before conjugation with GNPs, IgG was dialyzed against a 1000-fold volume of 10 mM Tris-HCl buffer, pH 9.0, for 2 h at 4–6 °C. Then, 0.1 M K_2_CO_3_ was added to the GNP1–GNP6 solutions (OD = 1.0 at the peak wavelength) until a pH of 9.0 was reached. After reaching the same pH level equal to 9.0, the GNP solutions were added to the IgG solutions with stirring. The mixtures were incubated for 30 min at 20–22 °C and stirred, after which an aqueous solution of PEG-SH was added to a final concentration of 0.25%. Conjugates of GNPs with IgG were centrifuged twice using an Allegra 64R centrifuge (Beckman Coulter; Indianapolis, IN, USA) for 15 min at 4 °C and accelerations of 25,000× *g* (GNP1–IgG), 22,000× *g* (GNP2–IgG), 20,000× *g* (GNP3–IgG), 15,000× *g* (GNP4–IgG), 12,000× *g* (GNP5–IgG), and 10,000× *g* (GNP6–IgG). 

The supernatants from the second centrifugation were separated and used to determine the concentration of bound and unbound antibodies by measuring fluorescence, as described in the next section. The pellets were resuspended in 0.05 M phosphate buffer containing 0.1 M NaCl and 0.25% PEG-SH, and preparations of GNP(1–6)–IgG conjugates with OD 5.0 were obtained.

### 2.6. Determination of the Antibody Number Bound to GNPs

The supernatants obtained after the centrifugation of GNP(1–6)–IgG conjugates were divided into 2 equal parts. IgG were added to the first half to reach a final concentration of 3.15 μg/mL to obtain calibration solutions; nothing was added to the other half. The resulting solutions were poured into 96-well plates, 200 μL per well (two replicates for each solution), and the fluorescence was measured (“Measurement of Fluorescence”). The difference in fluorescence for solutions with and without antibodies corresponds to the fluorescence of 3.15 μg/mL of antibodies. This value was used to calculate the concentration of antibodies in the supernatant, which in μg/mL corresponds to the value:

F ∗ C/(Fc − F)(1)

where F represents the fluorescence of the supernatant, Fc is the fluorescence of the supernatant with the addition of antibodies, and C is the concentration of added antibodies (in our experiments, C = 3.15 μg/mL).

The difference between the antibody concentration added to the reaction solution and the antibody concentration in the supernatant provides the antibody concentration in the corresponding conjugate with GNPs.

### 2.7. Determination of the Number of Reactive Antibodies on the GNP Surface

Each GNP–IgG conjugate (OD 5.0) was divided into 7 portions of 1.5 mL each. In 6 portions, CRP solutions were added to the final CRP concentrations of 10, 8, 6, 4, 2, and 1 μg/mL. No CRP was added to the seventh portion. The mixtures were then incubated for 30 min at 20–22 °C with stirring and then centrifuged twice at the aforementioned accelerations. The supernatant from the second centrifugation was then used to determine the number of bound and unbound CRP in the same way described for determining bound and unbound antibodies.

### 2.8. Fluorescence Measurement

Protein fluorescence spectra were recorded on a Perkin Elmer EnSpire 2300 microplate reader (Waltham, MA, USA). Spectra were measured in Nunc MaxiSorp white microplates (Roskilde, Denmark) at an excitation wavelength of 280 nm in the emitted light wavelength range of 290–500 nm.

## 3. Results

### 3.1. Method for Determining the Composition of Protein–GNP Conjugates by Intrinsic Protein Fluorescence

The method for determining the composition of GNP conjugates with antibodies and their antigen-binding activity is based on measuring tryptophan fluorescence twice: in solutions obtained after the conjugates’ centrifugation and in the same solutions after adding a known amount of protein (antibody or antigen) to them.

**Figure 1:**
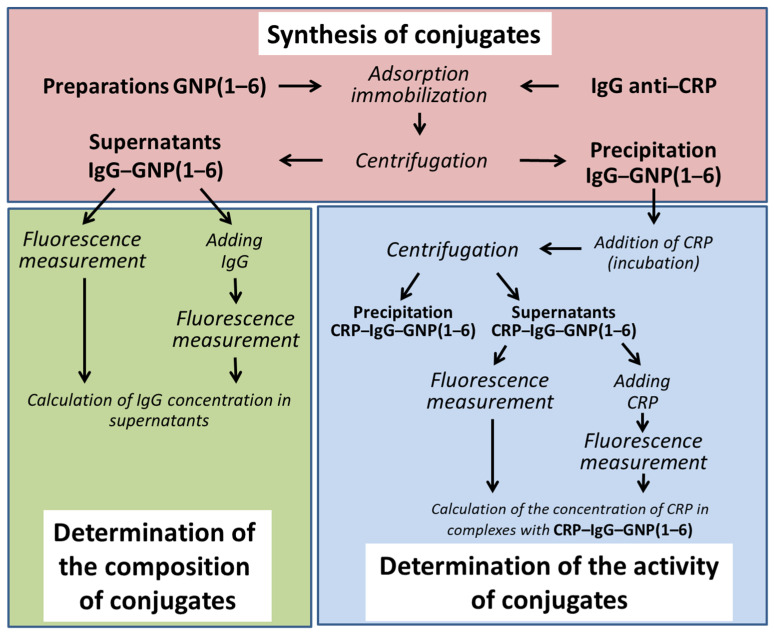
Schematic of the experiment to determine the number of antibodies immobilized on the GNP surface.

The diagram in Figure 1 demonstrates 3 parts of the experiment: 1. synthesis of conjugates, 2. determination of their composition, and 3. determination of antigen-binding capacity. The sequence of actions taken to determine the composition and activity of conjugates is identical. In the first case, GNPs are incubated with antibodies and centrifuged to separate complexes with nanoparticles; the supernatants are divided into 2 parts, and an additional portion of antibodies of a known concentration is added to one-half of the supernatants. In the second case, GNP–antibody conjugates are incubated with the antigen and centrifuged; the supernatants are then divided into 2 parts, and an antigen of a known concentration is added to one-half of the supernatants.

Tryptophan fluorescence depends on numerous factors, including the composition of the immediate environment. Even small changes in the ionic composition of the solution and protein conformation can significantly affect the signal [33,42,43]. For this reason, we used supernatants after conjugate centrifugation to obtain calibration solutions, to which known amounts of protein were added. The increase in fluorescence in the solution after the addition of this portion of the protein was used as a calibration signal. This approach makes it possible to achieve the identical ionic environment of fluorescent molecules in tested samples and calibration solutions.

### 3.2. Characterization of GNP Preparations

**Figure 2:**
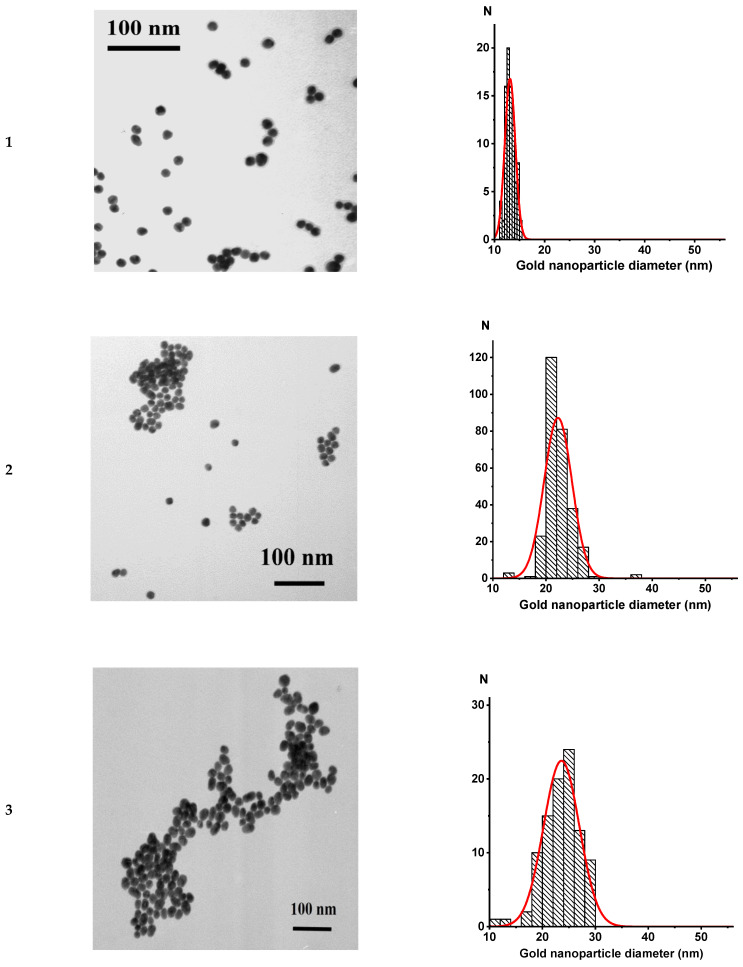

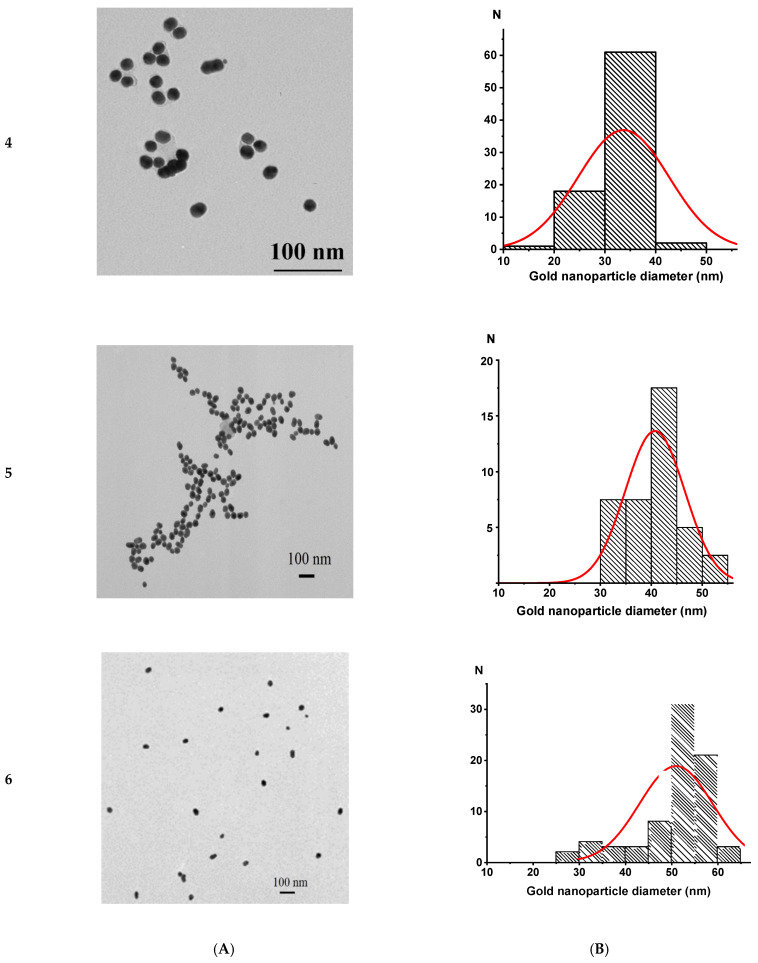
Electron micrographs (**A**) of preparations GNP1 (**1**), GNP2 (**2**), GNP3 (**3**), GNP4 (**4**), GNP5 (**5**), and GNP6 (**6**) and diameter distribution histograms (**B**). Sample sizes (N) were 60–90.

The sizes and homogeneity of the GNPs were characterized by TEM according to the previously described method [13]. The shape of GNPs in all 6 preparations was close to spherical, and the preparations did not contain aggregates (Figure 2). The average diameters were determined from images of the nanoparticles, per 60–90 measurements (Table 1).

**Figure 3:**
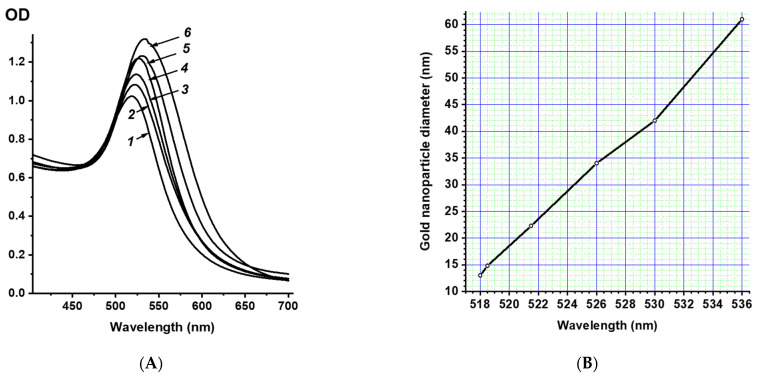
Absorption spectra (**A**) of the preparations GNP1 (**1**), GNP2 (**2**), GNP3 (**3**), GNP4 (**4**), GNP5 (**5**), and GNP6 (**6**) and the calibration dependence (**B**) of the maximum of the absorption spectrum on the diameter of the GNP.

The synthesized GNP preparations were also characterized spectrophotometrically. Spherical GNPs have a characteristic absorption peak at wavelengths of 515–540 nm, which is associated with the surface plasmon resonance effect arising from the resonance between frequency of collective oscillations of free electrons on the GNP surface and that of the light wave [44]. Figure 3 shows the spectra of the GNP1–GNP6 preparations. With an increase in the GNP diameter, a shift in the absorption spectra maxima of their solutions to the long-wavelength region was observed.

The GNP diameters were estimated using the calibration dependence of the absorption spectrum maximum on the GNP diameter, which we obtained earlier [41] and are shown in Figure 3B. For GNP1–GNP6 preparations, the average diameters were 14, 23, 28, 35, 42, and 55 nm according to this calibration dependence, respectively. The data obtained using the two methods were in good agreement, and the differences in the determined dimensional parameters were only 2–6%.

### 3.3. Synthesis of Antibody Conjugates with GNPs

When synthesizing GNPs, we took the same amount of chloroauric acid (0.01% in final volume) for all preparations. Only the concentration of the reducing agent was varied. With an increase in the particles’ diameter, surface area also increases as a square of the diameter, but the number of particles in the solution decreases in proportion to the diameter in the cube. Thus, the total sorption surface area decreases. Therefore, to obtain conjugates with GNPs of different sizes, we used different immunoglobulin G (IgG) concentrations, calculated so that one IgG molecule in all preparations had the same sorption surface area. This number was selected based on the concept of the protein globule’s average size and the average surface area occupied by antibodies during monolayer immobilization.

Earlier, when considering the sorption of IgG on the GNP surface, estimates of the number of antibodies in monolayer immobilization were proposed [11,13,14,32]. Based on data from different articles, we recalculated the area occupied by one IgG molecule. According to various studies, the IgG molecule occupies from 15 to 36 nm^2^ of the GNP surface. Note that the minimum score is achieved when the orientation of the immobilized IgG molecule is not random and/or the angle between Fab fragments of the IgG molecule is reduced to a minimum, i.e., for an energetically unstable conformation. On the other hand, at the maximum estimate, a convex shell of a branched IgG structure was considered, which does not correspond to the maximum dense surface coverage. Given these constraints, we preceded the calculations given below using the middle value for surface coverage by one IgG molecule being equal to 25 nm^2^. The corresponding numbers of antibodies to fill the monolayer for each GNP preparation are shown in Table 2. Thus, the six conjugate–antibody preparations were obtained with equal loading in terms of the nanoparticles’ surface area.

### 3.4. Calculation of the Numbers of Immunoglobulin G Sorbed on GNPs

**Figure 4:**
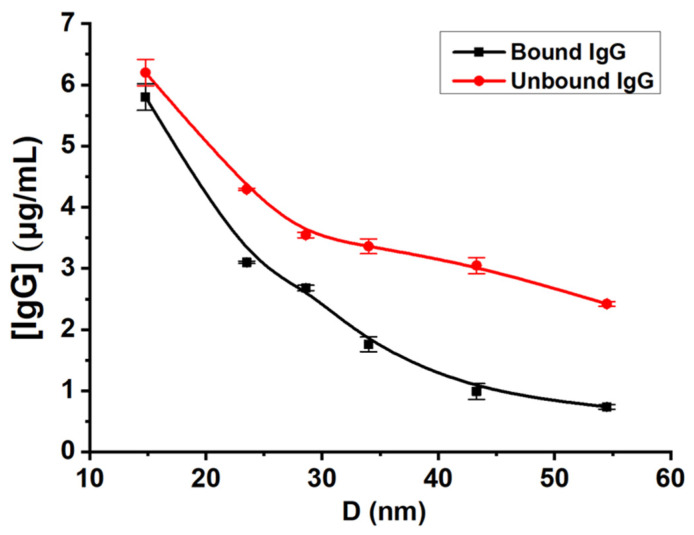
Calculated concentrations of immunoglobulins in supernatants and in conjugates (*y*-axis) for GNPs of different diameters (*x*-axis).3.5. Determination of the Antigen-Binding Activity of Antibodies after Conjugation.

Based on the registered fluorescence spectra (Supplementary Materials Figure S1), difference in fluorescence in the tested samples and calibration solutions and on that of calculating the amount of GNPs in the solution, the amount of protein in the conjugates was calculated (“Materials and Methods”). The obtained values of the fluorescence intensity were used to calculate the concentrations of the sorbed IgG in conjugates with nanoparticles. The amounts of IgG that were free or bound with GNPs are summarized in Supplementary Materials Table S1. Figure 4 shows the processing of the calculated concentrations of bound and free antibodies for particles of different sizes. These data demonstrate the smaller particles collectively bind more protein than the larger ones, although the added amount of IgGs per unit surface area of the particles did not differ among preparations.

It should be noted that the pH value equal to 9.0 being chosen for IgG immobilization accords to common recommendations for preparation of GNP-IgG conjugates for analytical purposes [45]. Early and recent studies [22,46,47,48] repeatedly confirm that this pH provides reaching both colloidal stability and high antigen-binding efficiency of the obtained products. The impact of pH to composition of conjugates was indicated earlier [32], and the technique for conjugates characterization presented in this paper may be used to evaluate this impact as well as impact of other parameters of the reaction media and properties of interacting molecules.

Thus, works [49,50] demonstrated that the ratio of IgG molecules, differently oriented on the surface, depends on the pH and ionic strength of the reaction medium, the composition and state of the adsorbing surface, temperature, and other factors, as well as on the surface density of bound IgG. However, the contribution of each factor to the final result requires additional studies using a wide variety of methods.

For the nanoparticle-protein complexes, the existence of protein subpopulations has been demonstrated, which are characterized by different times of dissociation of their bonds with the nanoparticle. It was shown that during the sorption of proteins on nanoparticles, the first portions of the protein firmly bind to the surface, forming a “hard” corona. After filling the surface, protein sorption continues over the first layer, and a “soft” corona of weakly bound protein molecules is formed [51,52]. The characteristic time of desorption of molecules of the “soft” corona is several hours, while the “hard” corona is stable for a long time [53,54]. Some conjugates of proteins with nanoparticles have been shown to retain activity for more than six months [55]. Thus, the stability of the conjugates directly depends on their composition. In our study, to obtain the IgG-GNP conjugates, we used IgG concentrations that did not exceed the filling of the monolayer (conditions for the formation of a “hard” corona). This allows expecting long-time stability of the obtained conjugates. Note that in biotechnological practice, when prolonged storage of nanoparticle-protein conjugates is required, various stabilizing components are added to the initial medium used in the synthesis [56], which further reduces the dissociation of the conjugates.

### 3.5. Determination of the Antigen-Binding Activity of Antibodies after Conjugation

**Figure 5:**
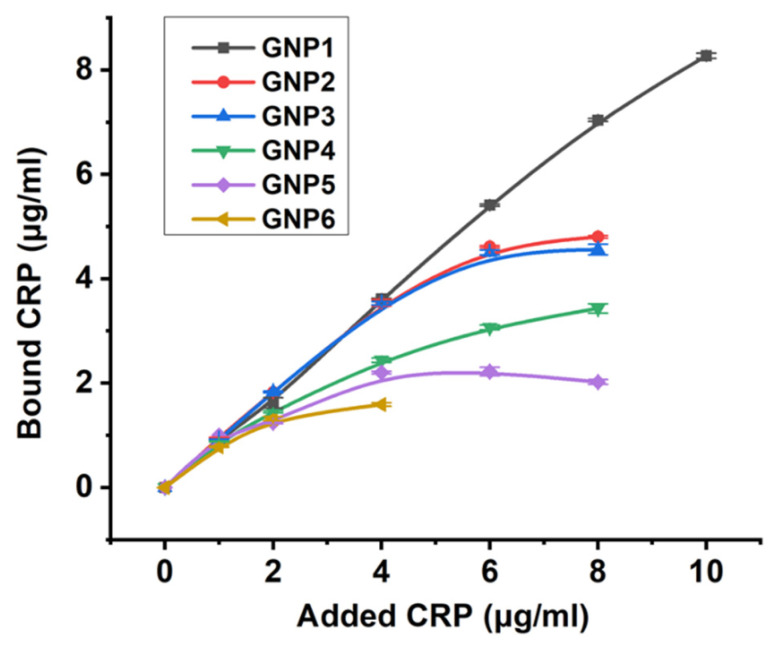
Calculated concentrations of CRP bound to conjugates of GNPs and antibodies (*y*-axis) for conjugates of different diameters (*x*-axis).

The amounts of CRP bound to the GNP conjugates are shown in Figure 5 (the spectra of CRP in supernatants are shown in Supplementary Materials Figures S2–S7). With the exception of GNP1 (14.8 nm), saturation was observed for all preparations at antibody concentrations of 4–6 μg/mL. Based on the obtained data on the maximum amount of antigen bound by the conjugates, the percentages of active antigen-binding valences in the conjugates were calculated as shown in Table 3 (initial data and intermediate calculations are given in Supplementary Materials Table S2). Per these results, 17–34% of the valences in the conjugates can bind antigen. For 14.8 nm nanoparticles, the result was somewhat underestimated because we did not reach saturating concentrations of the antigen. Therefore, the most reliable results were obtained for GNP2–GNP6, which demonstrate the retention of the activity for 21–34% of antigen-binding sites.

For one GNP preparation (GNP2, average diameter of 23.5 nm), the effect of the number of antibodies in the conjugate on the percentage of active antigen-binding sites was investigated. To do so, the GNP2–IgG conjugates were synthesized with loads of 7.4, 3.7, and 1.85 μg/mL IgG, which theoretically should correspond to a full monolayer, one-half of a monolayer, and one-quarter of a monolayer, respectively. The results obtained after analyzing the composition of the conjugates are presented in Table 4.

When adding one antibody molecule per 100 nm^2^ of the nanoparticles’ surface area, the antibodies were absorbed almost completely (the supernatant fluid’s fluorescence signal did not differ reliably from the background). When the concentration of IgG was increased and one molecule accorded to 25 nm^2^ of the gold surface, about 40% of the antibodies remained in the supernatant.

The IgG–GNP interaction in our study does not involve any components promoting oriented immobilization. Thus, the orientation of IgG could be assumed as random, but direct verification and statistical confirmation of completely random orientation requires further studies beyond this work.

**Figure 6:**
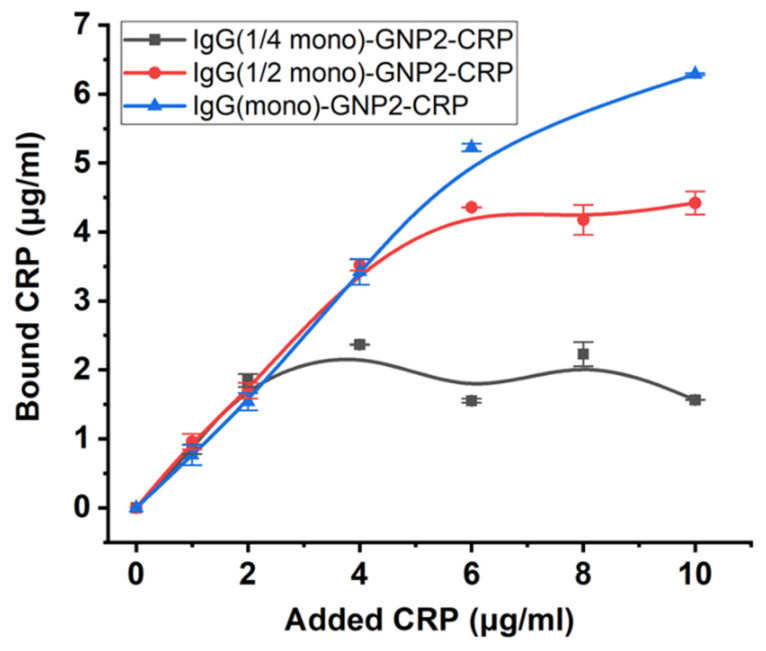
CRP binding to conjugates of GNPs with 23.5 nm diameter containing 5.1, 2.6, and 1.85 μg/mL of bound antibodies. Dependence of bound CRP concentrations (*y*-axis) on added CRP concentrations (*x*-axis).

To synthesized and purified conjugates with different antibody content, CRP was added at concentrations of 1–10 μg/mL and incubated, and then nanoparticles were separated by centrifugation twice. The supernatants obtained in this way were analyzed to determine the free CRP content (the spectra of CRP in supernatants are shown in Supplementary Materials Figures S8–S10), and the CRP content in complexes with conjugates was calculated from the material balance (Figure 6 and Supplementary Materials Table S3).

The obtained values of the percentage of active antigen-binding sites did not reveal significant differences for the conjugates of different compositions (Table 5). However, because adding less antibodies during synthesis led to more efficient nanoparticle binding (Table 4), it seems preferable to use conjugates with a lower antibody–GNP ratio at a higher concentration because doing so minimizes antibody loss.

### 3.6. Comparison of the Obtained Parameters with Earlier Published Ones

The results obtained in this study are in good agreement with other studies’ results and expand on those results significantly. For example, using the enzyme-linked immunosorbent assay (ELISA), the composition and activity of conjugates of GNPs with anti-species antibodies were studied. It was found that when antibodies were immobilized on GNPs with a diameter of 20.7 nm, taking into account the bivalence of IgG, only 12% of the valences of antibodies retained their antigen-binding ability. That is, the amount of IgG bound in the second layer was 24% of the amount of anti-species IgG sorbed in the first layer on the GNPs’ surface [29]. Based on ELISA, this technique provides reliable results but is very laborious. Therefore, a single conjugate was analyzed in this way. A group of researchers led by Driskell used a similar approach to analyze antibody conjugates against peroxidase with GNPs with a diameter of 63.4 nm; they found the percentage of active antibodies during adsorption immobilization was 23%. Using affinity immobilization via staphylococcal Ig-binding protein A increased the percentage of active antibodies to 91% [28]. In a subsequent study, it was shown that by varying the pH from 7.5 to 8.5, the percentage of active antibodies changed from 33 to 18% [30]. In addition, the data presented are consistent with Kaur and Forrest’s studies on the shift of GNPs’ local surface plasmon resonance peak upon conjugation with proteins [11]. For example, they demonstrated that approximately 100 IgGs are sorbed on GNPs with a diameter of 50 nm (according to our data, 84.9 molecules per a GNP with a diameter of 54.5 nm).

When IgG molecules are immobilized on a surface, four fundamentally different orientations are possible that are classified in [57] as side-on (one Fc and one Fab attached to the surface), tail-on (Fc attached to the surface), head-on (both Fabs attached to the surface), and flat-on (all three fragments attached to the surface). Although the quantitative ratio between these variants remains unclear, it should be noted that only the tail-on orientation provides full accessibility of antigen-binding sites. This factor explains the fact that most of the antibodies’ valencies lose their binding capacities after immobilization.

Moreover, the steric factor makes its impact. When working with a large antigen (such as C-reactive protein, mm 125 kDa, used in this study), one bound antigen molecule can shield (make inaccessible) neighboring IgG valences.

However, other studies’ results differ from our data. Zhang et al. [27] investigated the conjugation of fluorescein-labeled IgG with GNPs with a diameter of 14.8 nm via 3 methods: measuring the conjugate’s fluorescence after dissolving the GNP with sodium cyanide, measuring the supernatant’s fluorescence after separating the conjugate by centrifugation, and using ELISA; the values of 3.9, 13.3, and 9.9 bound IgG per one nanoparticle were obtained, respectively. The researchers also found 4.6 molecules of anti-species antibodies labeled with peroxidase could be bound per one particle of the conjugate. The results of the second approach wholly coincide with our data, whereas others produced fundamentally different results. Different approaches used to acquire the measurements may have caused these discrepancies.

The concept of the formation of a protein shell during sorption on nanoparticles is still incomplete. This indicates the need for new data on the quantitative regularities of the process and the development of new approaches to obtain them.

## 4. Conclusions

Conjugates of antibodies with GNPs are one of the most commonly used labeled reagents in biochemical studies and analytical systems. However, questions about their composition and binding capacity remain unanswered because of the limitations of the analytical methods used. The proposed method for determining these parameters is based on measuring the intrinsic fluorescence of proteins and is characterized by high productivity and accuracy to find the best conjugate composition for practical application. Because of the preliminary separation of the conjugates from the analyzed solutions, the influence of nanoparticles on fluorescence is excluded, which increases the reliability of the analysis. This technique was used for GNP conjugates with antibodies against CRP; however, the approach remains universal and can be used for other protein antigen–antibody pairs and nanoparticles. The primary criteria for the described technique’s applicability are the presence of tryptophans in the studied protein’s composition and the possibility of nanoparticles’ complete removal from the solution after conjugation with proteins.

The study showed that after the synthesis of antibody conjugates with GNPs, only 17–34% of antigen-binding sites retained the ability to bind antigen (taking into account the bivalence of IgG). An increase in the nanoparticle size typically leads to a similar increase in the proportion of active antibodies in the conjugate. However, the total amount of adsorbed antibodies (per mL of colloidal solutions, normalized by optical density) sharply decreases with an increase in particle diameter due to a decrease in the particles’ total surface area. A decrease in the concentration of antibodies during synthesis leads to an increase in the proportion of sorbed antibodies but does not change the availability of active valences for binding with the antigen. Sorption of antibodies on GNPs in the ratio of one molecule per 100 nm^2^ of the surface leads to the nearly complete binding of antibodies. From the viewpoint of efficient antibody consumption, it is preferable to use antibody concentrations not exceeding this ratio for sorption.

Because complexes of antibodies with GNPs are often used as marker agents in immunoanalytical systems, the results of this study can be useful for targeted optimization of the composition of nanoconjugates to improve the metrological parameters of analyses. For example, it follows from the data obtained that to increase the binding capacity of conjugates and efficient consumption of reagents, it is better to use preparations with a lower antibody–nanoparticle ratio but at a higher concentration than lower concentrations of conjugates with a large number of sorbed antibodies.

## Figures and Tables

**Table 1:** Dimensional and spectral characteristics of GNPs.

No	Absorption Maximum, nm	GNP Diameter According to Spectral Data, nm	GNP Diameter According to TEM Data, nm	Ellipticity
1	518	14	14.8 ± 1.1	1.18 ± 0.29
2	521	23	23.5 ± 1.5	1.19 ± 0.21
3	523	28	28.6 ± 2.3	1.22 ± 0.36
4	527	35	34.0 ± 2.5	1.25 ± 0.31
5	530	42	43.3 ± 2.9	1.26 ± 0.32
6	534	55	54.5 ± 7.9	1.65 ± 0.53

**Table 2:** Parameters of GNPs and the calculated amounts of IgG added to fill the monolayer.

No	GNP Diameter, nm	GNP Surface Area, nm^2^	IgG Number Per One GNP (Monolayer, Theoretically), pcs	[GNP], pcs/mL	[IgG] Monolayer, pcs/mL	[IgG] Monolayer, μg/mL
1	14.8	688	28	1.77 × 10^12^	49.6 × 10^12^	12.0
2	23.5	1734	69	4.43 × 10^11^	30.6 × 10^12^	7.40
3	28.6	2568	103	0.25 × 10^12^	25.8 × 10^12^	6.23
4	34.0	3630	145	1.46 × 10^11^	21.1 × 10^12^	5.12
5	43.3	5887	235	0.71 × 10^11^	16.7 × 10^12^	4.04
6	54.5	9327	373	0.35 × 10^11^	13.1 × 10^12^	3.16

**Table 3:** Calculated numbers of antigen-binding sites on one conjugate particle and their binding capacity.

GNP Average Diameter, nm	IgG Per One GNP	IgG Valences in the Conjugate, nM	Bound CRP Max, nM	Active Valences, %
14.8	13.2	379.1	66.2	17.5
23.5	28.1	191.4	41.4	21.6
28.6	43.0	156.7	36.5	23.3
34.0	77.0	153.0	33.8	22.1
43.3	56.0	53.7	17.8	33.1
54.5	84.9	37.4	12.7	34.0

**Table 4:** Results of the study of the composition of GNP2–IgG conjugates.

IgG Added, μg/mL	IgG in Supernatant, μg/mL	IgG in Conjugate, μg/mL	Bound IgG, %
7.4	2.9	4.5	60.5
3.7	1.0	2.7	72.6
1.85	0	1.85	100

**Table 5:** Results of the study of the CRP binding by the GNP2–IgG conjugates.

IgG in the Conjugate, μg/mL	Concentration of Antigen-Binding Sites in the Conjugate, nM	Maximum Bound CRP, nM	Active Antigen-Binding Sites, %
4.5	276	50.3	18
2.7	166	35.4	21
1.85	114	19.0	17

## Data Availability

Data are contained within the article. Initial data of instrumental measurements are available on request from the corresponding author.

## Supplementary Materials

The following are available online at https://www.mdpi.com/article/10.3390/nano11113117/s1, Figure S1: Fluorescence spectra of supernatants of conjugates GNP1–IgG (A), GNP2–IgG (B), GNP3–IgG (C), GNP4–IgG (D), GNP5–IgG (E), and GNP6–IgG (F), Figure S2: Fluorescence spectra of supernatants of the GNP1–IgG conjugate titrated with CRP at concentrations of 10 (A), 8 (B), 6 (C), 4 (D), 2 (E), 1 (F), and 0 (G) µg/mL, Figure S3: Fluorescence spectra of supernatants of the GNP2–IgG conjugate titrated with CRP at concentrations of 10 (A), 8 (B), 6 (C), 4 (D), 2 (E), 1 (F), and 0 (G) µg/mL, Figure S4: Fluorescence spectra of supernatants of the GNP3–IgG conjugate titrated with CRP at concentrations of 10 (A), 8 (B), 6 (C), 4 (D), 2 (E), 1 (F), and 0 (G) µg/mL, Figure S5: Fluorescence spectra of supernatants of the GNP4–IgG conjugate titrated with CRP at concentrations of 10 (A), 8 (B), 6 (C), 4 (D), 2 (E), 1 (F), and 0 (G) µg/mL, Figure S6: Fluorescence spectra of supernatants of the GNP5–IgG conjugate titrated with CRP at concentrations of 10 (A), 8 (B), 6 (C), 4 (D), 2 (E), 1 (F), and 0 (G) µg/mL, Figure S7: Fluorescence spectra of supernatants of the GNP6–IgG conjugate titrated with CRP at concentrations of 10 (A), 8 (B), 6 (C), 4 (D), 2 (E), 1 (F), and 0 (G) µg/mL, Figure S8: Fluorescence spectra of supernatants of the GNP2–IgG conjugate (monolayer) titrated with CRP at concentrations of 10 (A), 8 (B), 6 (C), 4 (D), 2 (E), 1 (F), and 0 (G) µg/mL, Figure S9: Fluorescence spectra of supernatants of the GNP2–IgG conjugate (1/2 monolayer) titrated with CRP at concentrations of 10 (A), 8 (B), 6 (C), 4 (D), 2 (E), 1 (F), and 0 (G) µg/mL, Figure S10: Fluorescence spectra of supernatants of the GNP2–IgG conjugate (1/4 monolayer) titrated with CRP at concentrations of 10 (A), 8 (B), 6 (C), 4 (D), 2 (E), 1 (F), and 0 (G) µg/mL, Table S1: Results of measuring the fluorescence of supernatant solutions after centrifugation of conjugates with GNPs(1-6) and calculated concentrations of IgG in solutions and in the conjugates, Table S2: Calculated concentrations of CRP bound to conjugates of GNPs (1–6) and CRP unbound (remaining in supernatants), Table S3: Calculated concentrations of CRP bound to conjugates of GNP2 with different contents of IgG.
