# Supplementary material for: Retention of Activity by Antibodies Immobilized on Gold Nanoparticles of Different Sizes: Fluorometric Method of Determination and Comparative Evaluation
Source: Nanomaterials (Basel). 2021 Nov 18;11(11):3117. doi: 10.3390/nano11113117 (PMC8625478; doi:10.3390/nano11113117)
Supplement: Supplementary file 1 [file nanomaterials-11-03117-s001.zip › nanomaterials-1444230-supplementary.pdf]

Supplementary Material

Retention of Activity by Antibodies Immobilized on Gold Nanoparticles of Different Sizes: Fluorometric Method of Determination and Comparative Evaluation

D. V. Sotnikov, N. A. Byzova, A. V. Zherdev, B. B. Dzantiev

A. N. Bach Institute of Biochemistry, Research Center of Biotechnology, Russian Academy of Sciences, 119071 Moscow, Russia

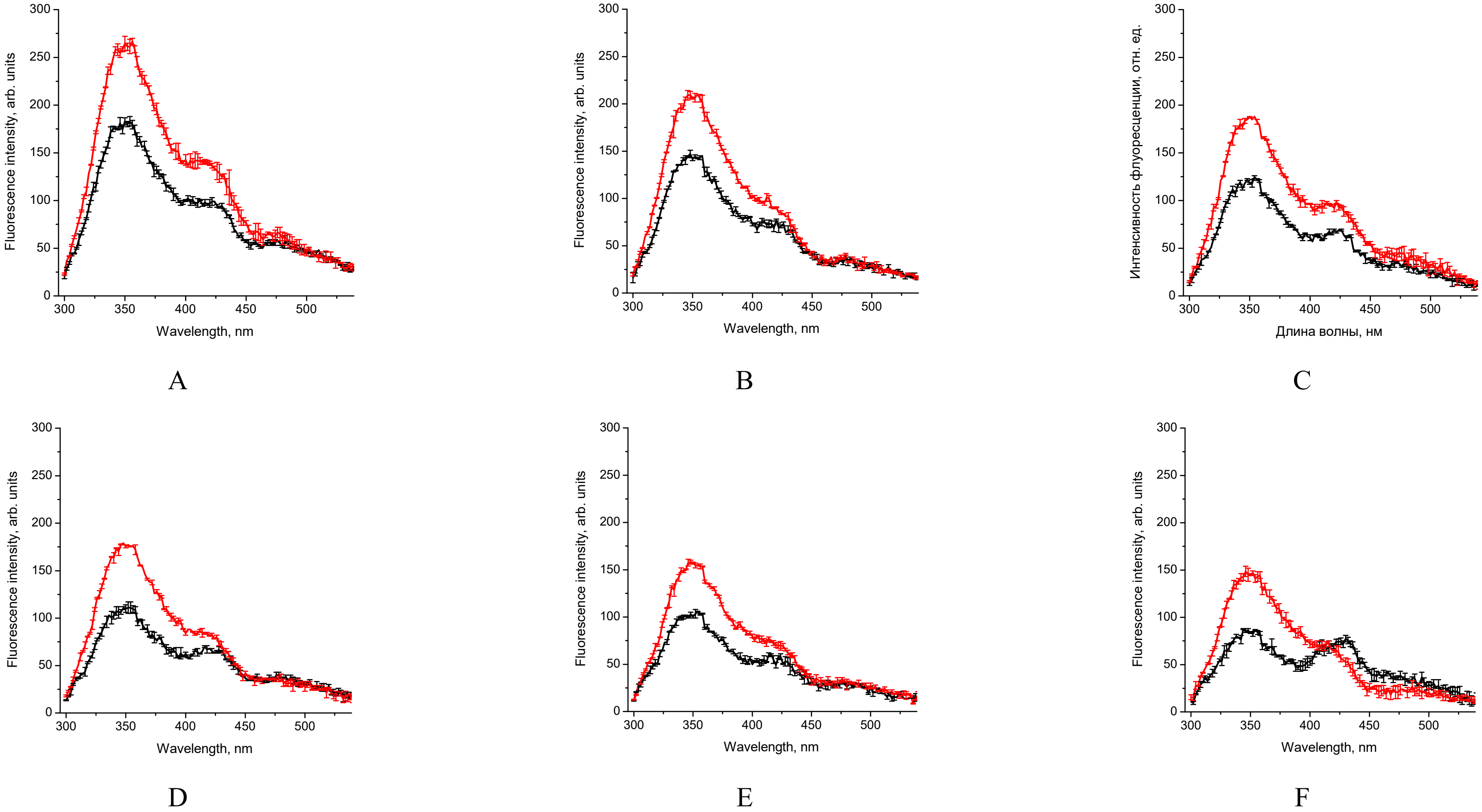

Figure S1. Fluorescence spectra of supernatants of conjugates GNP1–IgG (A), GNP2–IgG (B), GNP3–IgG (C), GNP4–IgG (D), GNP5–IgG (E), and GNP6–IgG (F). Red spectrum – after addition of 3.15 μg/mL IgG, black spectrum – without IgG.

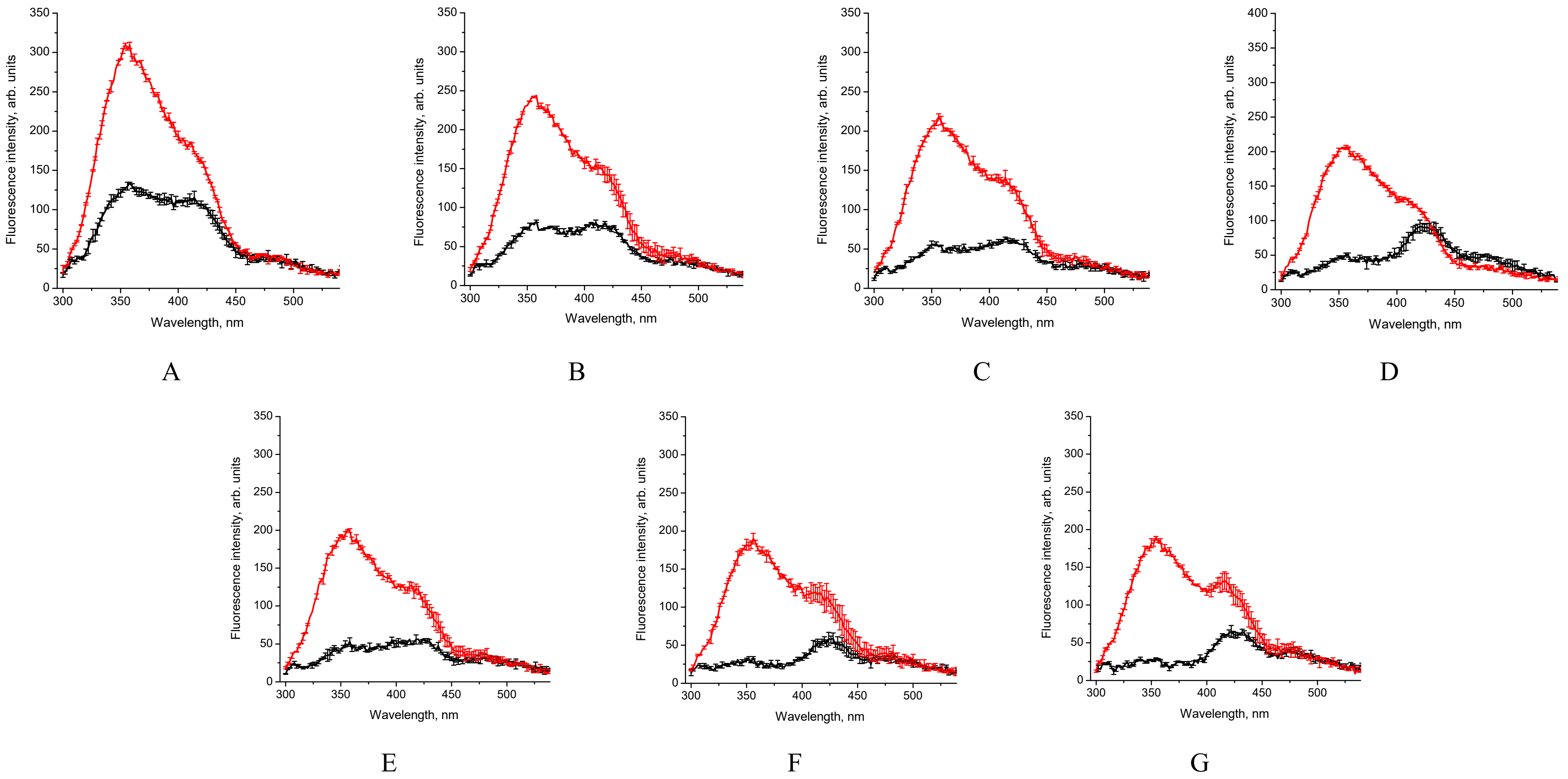

Figure S2. Fluorescence spectra of supernatants of the GNP1–IgG conjugate titrated with CRP at concentrations of 10 (A), 8 (B), 6 (C), 4 (D), 2 (E), 1 (F), and 0 (G) μg/mL. Red spectrum – after addition of 3.15 μg/mL CRP, black spectrum – without CRP.

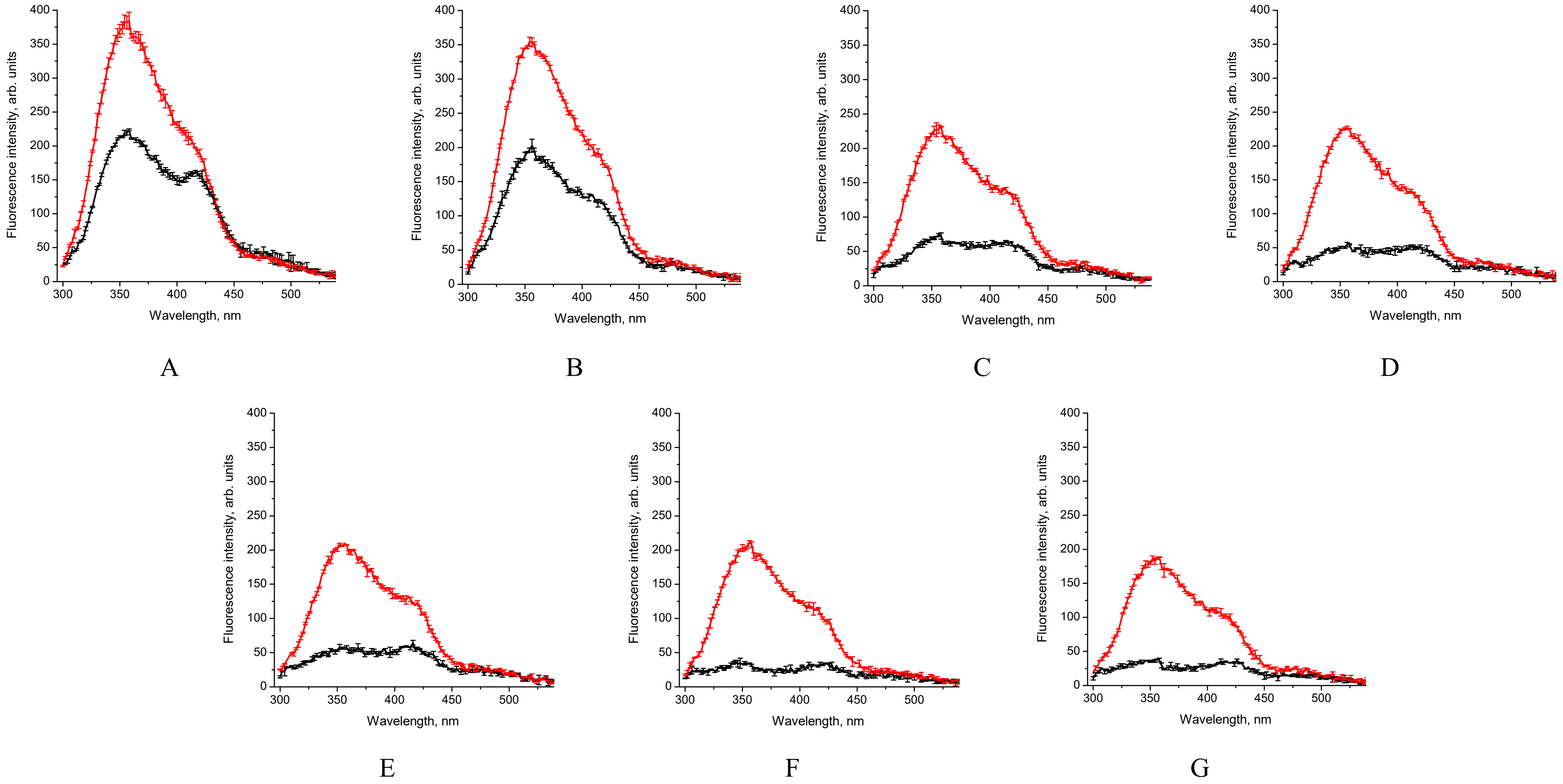

Figure S3. Fluorescence spectra of supernatants of the GNP2–IgG conjugate titrated with CRP at concentrations of 10 (A), 8 (B), 6 (C), 4 (D), 2 (E), 1 (F), and 0 (G) μg/mL. Red spectrum – after addition of 3.15 μg/mL CRP, black spectrum – without CRP.

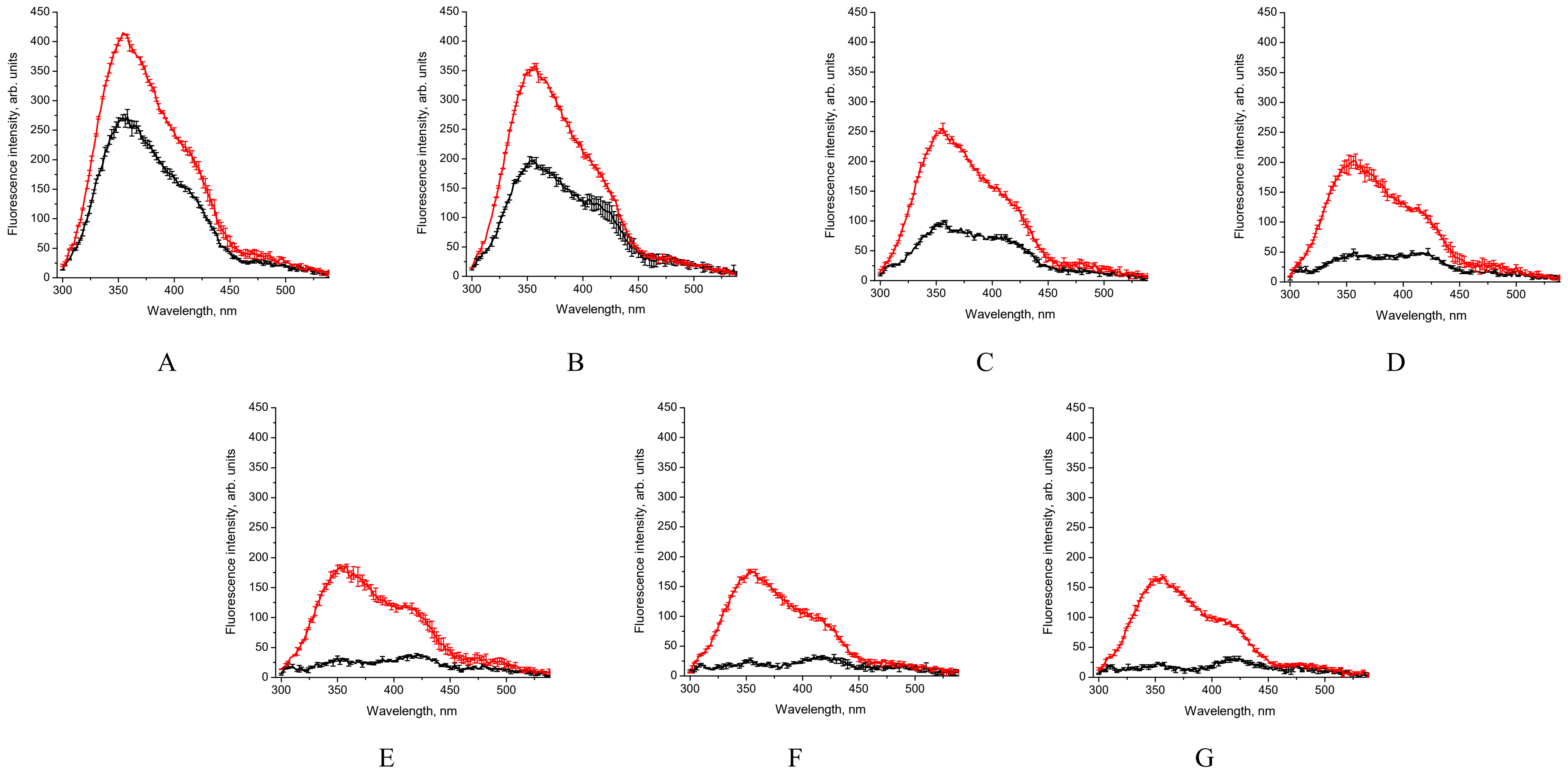

Figure S4. Fluorescence spectra of supernatants of the GNP3–IgG conjugate titrated with CRP at concentrations of 10 (A), 8 (B), 6 (C), 4 (D), 2 (E), 1 (F), and 0 (G) µg/mL. Red spectrum – after addition of 3.15 µg/mL CRP, black spectrum – without CRP.

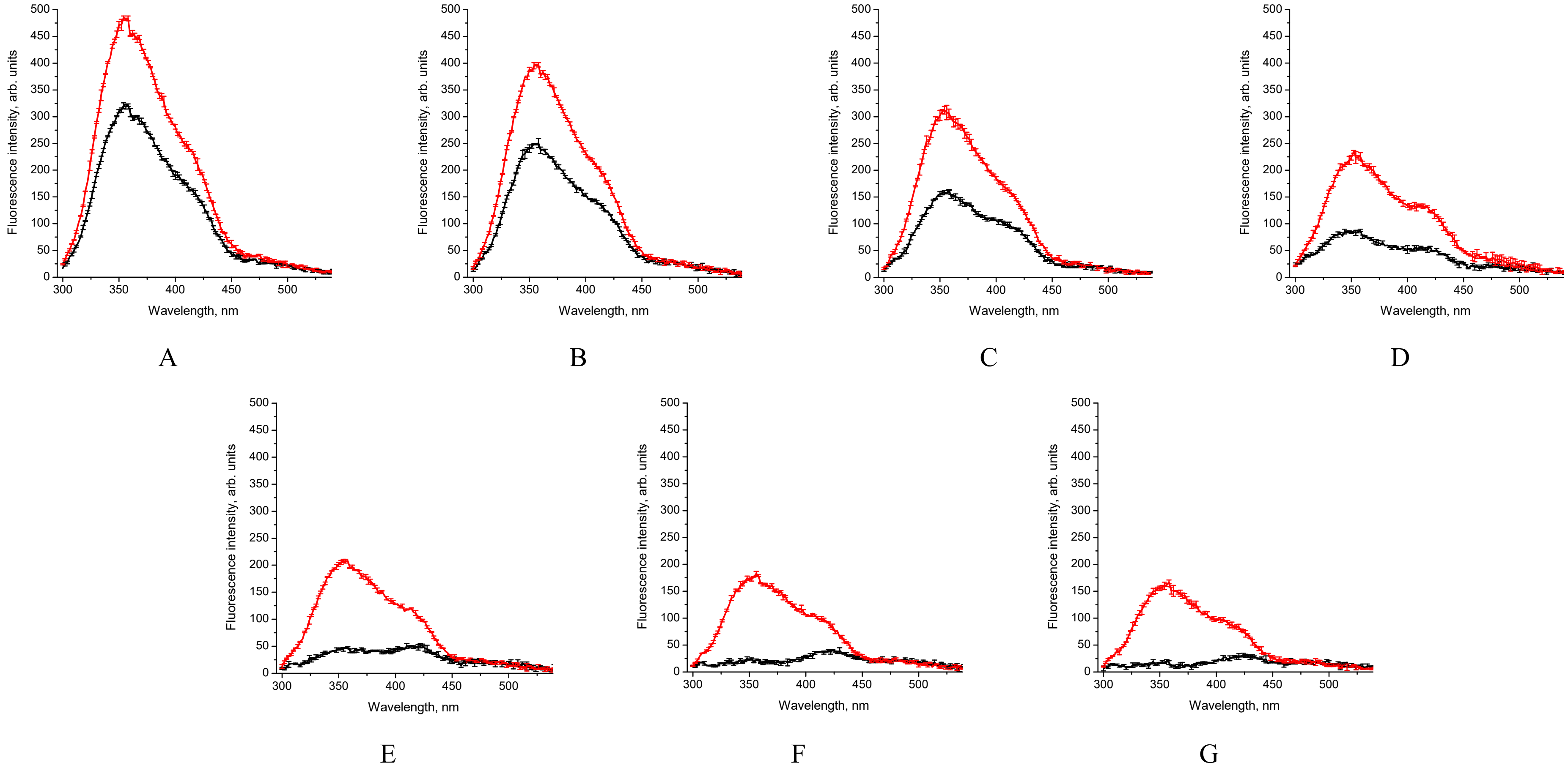

Figure S5. Fluorescence spectra of supernatants of the GNP4–IgG conjugate titrated with CRP at concentrations of 10 (A), 8 (B), 6 (C), 4 (D), 2 (E), 1 (F), and 0 (G) μg/mL. Red spectrum – after addition of 3.15 μg/mL CRP, black spectrum – without CRP.

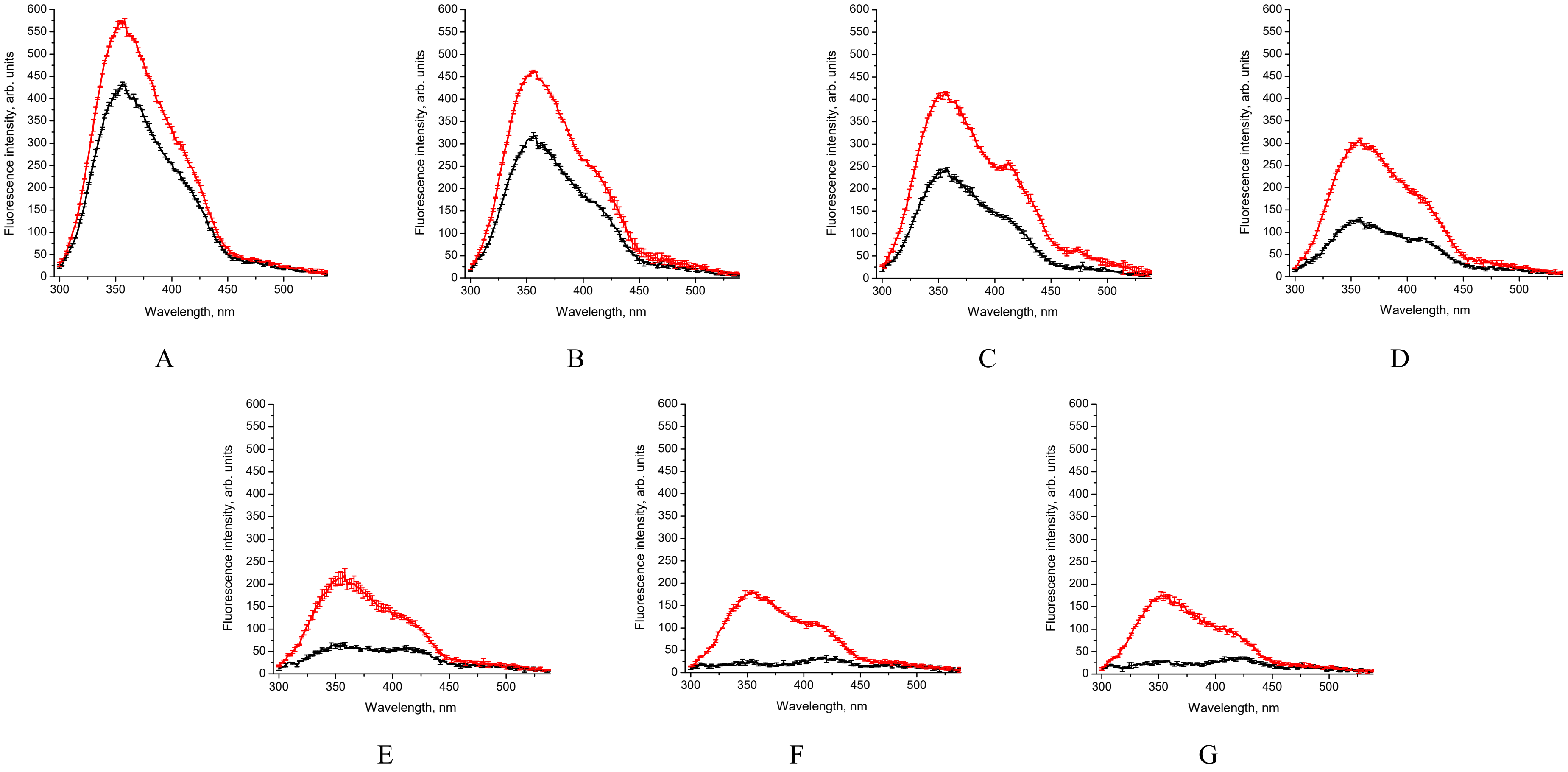

Figure S6. Fluorescence spectra of supernatants of the GNP5–IgG conjugate titrated with CRP at concentrations of 10 (A), 8 (B), 6 (C), 4 (D), 2 (E), 1 (F), and 0 (G) μg/mL. Red spectrum – after addition of 3.15 μg/mL CRP, black spectrum – without CRP.

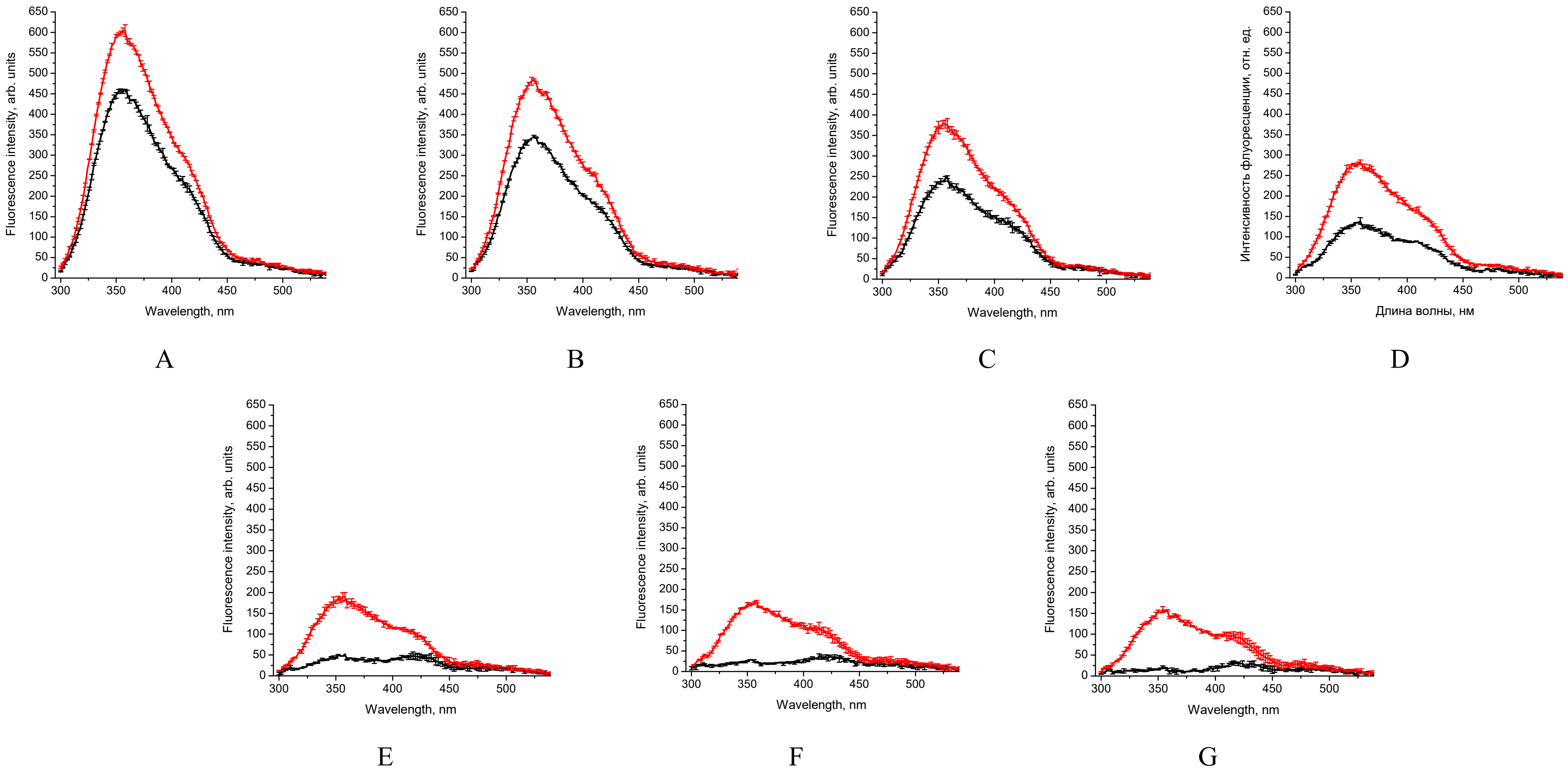

Figure S7. Fluorescence spectra of supernatants of the GNP6–IgG conjugate titrated with CRP at concentrations of 10 (A), 8 (B), 6 (C), 4 (D), 2 (E), 1 (F), and 0 (G) µg/mL. Red spectrum – after addition of 3.15 µg/mL CRP, black spectrum – without CRP.

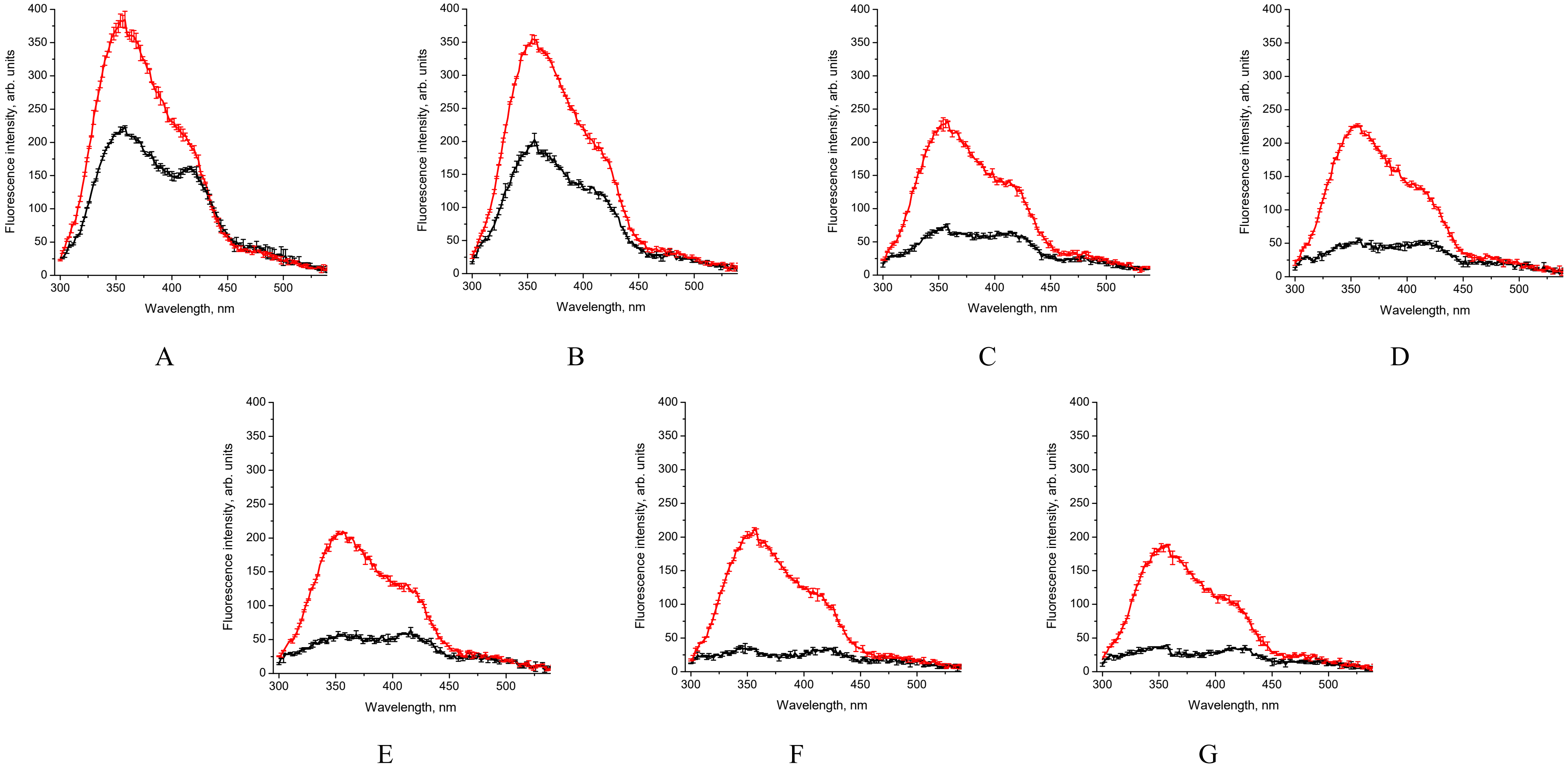

Figure S8. Fluorescence spectra of supernatants of the GNP2–IgG conjugate (monolayer) titrated with CRP at concentrations of 10 (A), 8 (B), 6 (C), 4 (D), 2 (E), 1 (F), and 0 (G) μg/mL. Red spectrum – after addition of 3.15 μg/mL CRP, black spectrum – without CRP.

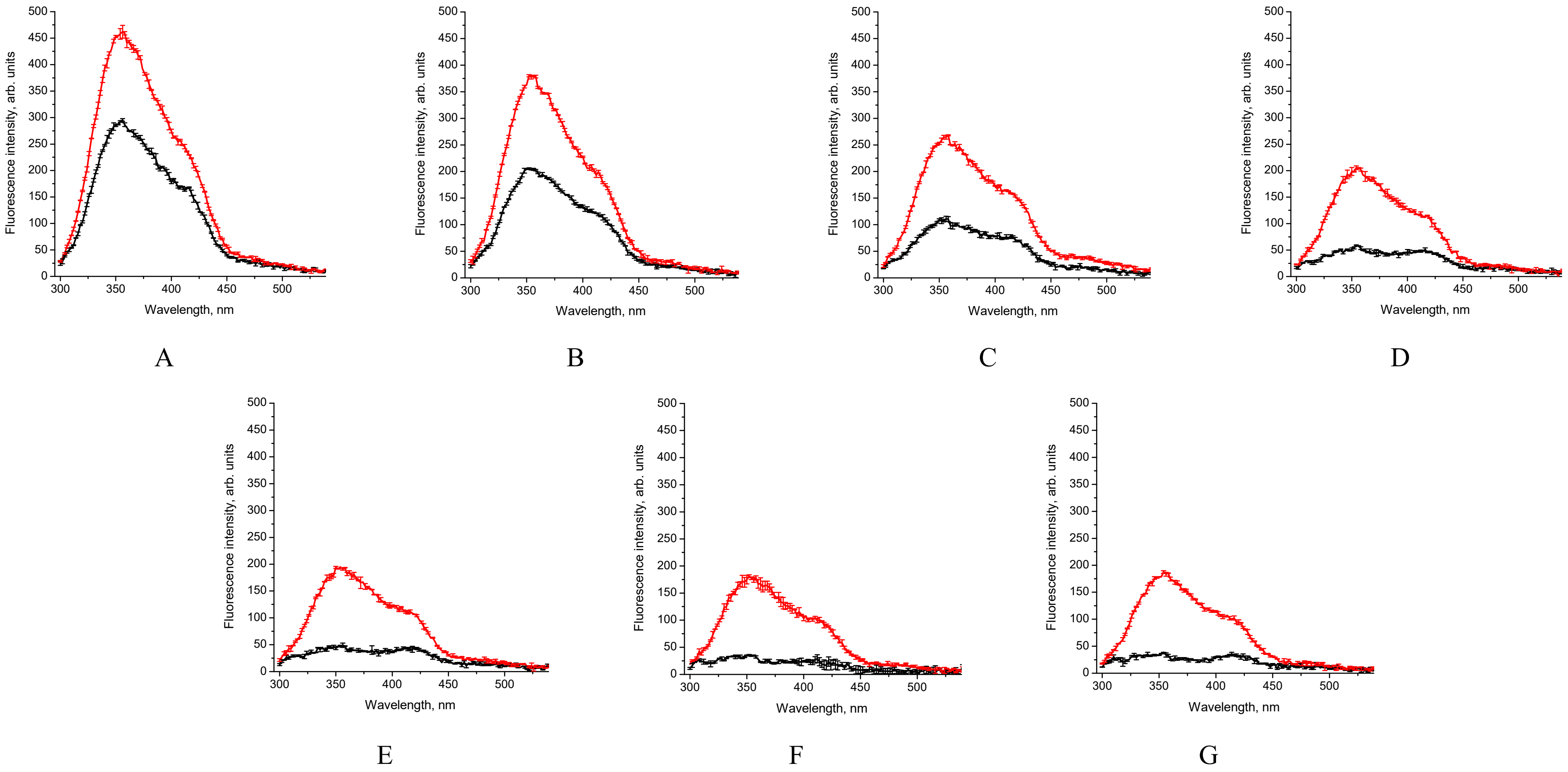

Figure S9. Fluorescence spectra of supernatants of the GNP2–IgG conjugate (1/2 monolayer) titrated with CRP at concentrations of 10 (A), 8 (B), 6 (C), 4 (D), 2 (E), 1 (F), and 0 (G) µg/mL. Red spectrum – after addition of 3.15 µg/mL CRP, black spectrum – without CRP.

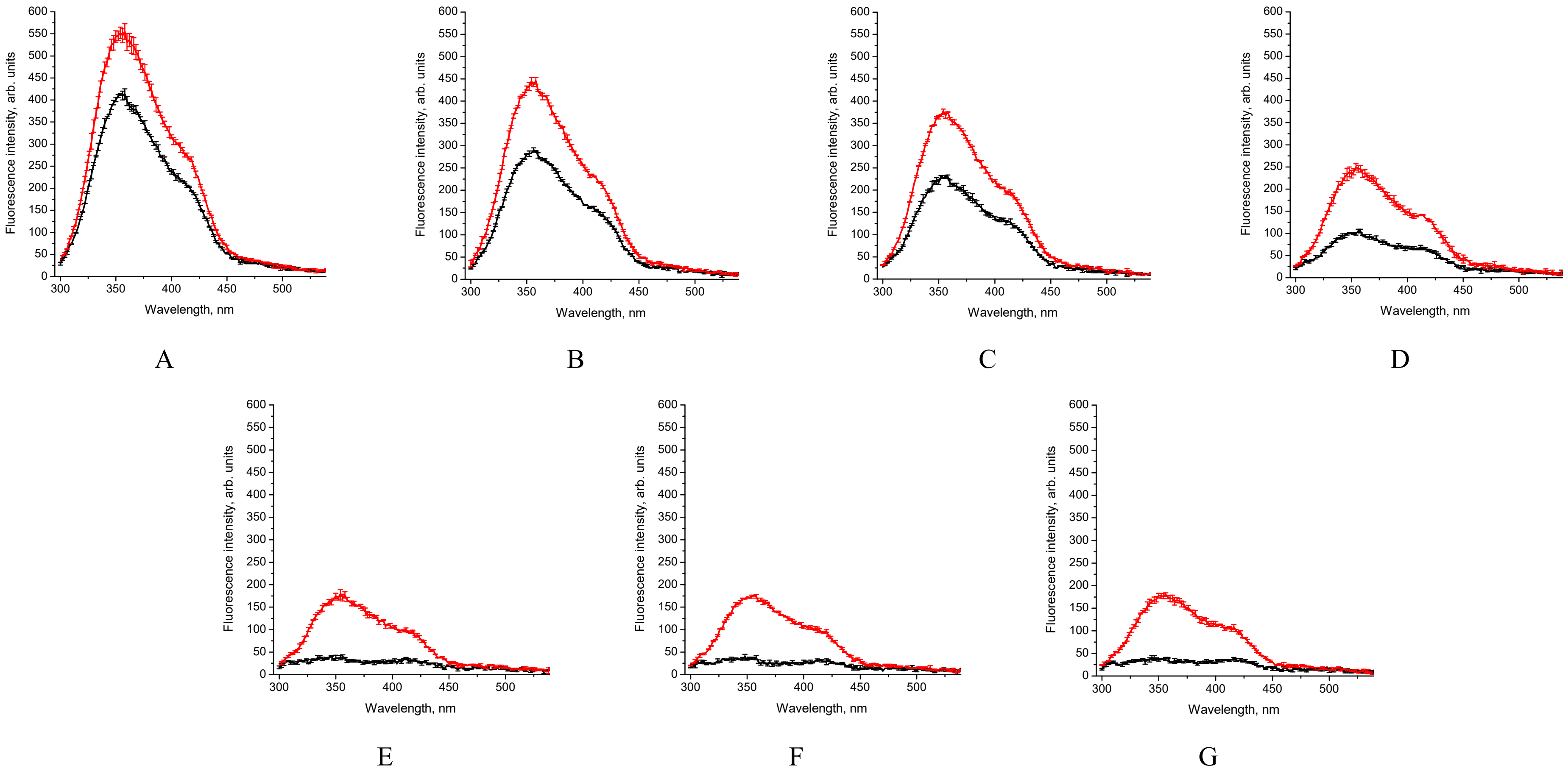

Figure S10. Fluorescence spectra of supernatants of the GNP2–IgG conjugate (1/4 monolayer) titrated with CRP at concentrations of 10 (A), 8 (B), 6 (C), 4 (D), 2 (E), 1 (F), and 0 (G) μg/mL. Red spectrum – after addition of 3.15 μg/mL CRP, black spectrum – without CRP.

Table S1. Results of measuring the fluorescence of supernatant solutions after centrifugation of conjugates with GNPs(1-6) and calculated concentrations of IgG in solutions and in the conjugates. Fluorescence signals are average fluorescence values at 350 nm for 2 replicates with subtracted background values for supernatants after centrifugation of pure GNPs(1-6) preparations stabilized with PEG-SH.

N	Particle diameter, nm	Added antibodies, µg/mL	Fluorescence in supernatants, arb. units	Error, %	Fluorescence in supernatants with added 3.15 µg/mL IgG, arb. units	Error, %	Calibration signal from 3, 15 µg/mL IgG, arb. units	IgG in supernatants, µg/mL	IgG in conjugates, µg/mL
1	14.8	12	144.7	3.5	224.4	3.2	73.5	6.2	5.8
2	23.5	7.4	108	1.1	172.2	1.3	79.2	4.3	3.1
3	28.6	6.2	85.1	1.2	150.8	0.3	75.6	3.5	2.68
4	34	5.1	76.1	3.6	143.2	0.3	71.3	3.4	1.76
5	43.3	4.0	71.4	1.8	125.3	1.2	73.8	3.0	0.99
6	54.5	3.2	55	1.5	115.6	2.7	71.6	2.4	0.74

Table S2. Calculated concentrations of CRP bound to conjugates of GNPs(1-6) and CRP unbound (remaining in supernatants).

	GNP1				GNP 2			
Added CRP, µg/mL	Bound CRP, µg/mL	Error, µg/mL	Unbound CRP, µg/mL	Error, µg/mL	Bound CRP, µg/mL	Error, µg/mL	Unbound CRP, µg/mL	Error, µg/mL
0	0	–	0	–	0	–	0	–
1	0.94	0.004	0.06	0.004	0.96	0.005	0.04	0.005
2	1.62	0.098	0.38	0.098	1.82	0.008	0.18	0.008
4	3.61	0.017	0.39	0.017	3.54	0.060	0.46	0.060
6	5.41	0.023	0.60	0.023	4.61	0.021	1.39	0.021
8	7.04	0.027	0.96	0.027	4.8	0.024	3.20	0.024
10	8.27	0.049	1.73	0.049	–	–	–	–

continues

	GNP3				GNP4			
Added CRP, µg/mL	Bound CRP, µg/mL	Error, µg/mL	Unbound CRP, µg/mL	Error, µg/mL	Bound CRP, µg/mL	Error, µg/mL	Unbound CRP, µg/mL	Error, µg/mL
0	0	–	0	–	0	–	0	–
1	0.92	0.008	0.08	0.008	0.88	0.007	0.12	0.007
2	1.83	0.017	0.17	0.017	1.45	0.019	0.55	0.019
4	3.53	0.031	0.47	0.031	2.44	0.040	1.55	0.040
6	4.5	0.045	1.46	0.045	3.07	0.046	2.93	0.046
8	4.56	0.102	3.44	0.102	3.43	0.089	4.57	0.089

continued

	GNP5				GNP6			
Added CRP, µg/mL	Bound CRP, µg/mL	Error, µg/mL	Unbound CRP, µg/mL	Error, µg/mL	Bound CRP, µg/mL	Error, µg/mL	Unbound CRP, µg/mL	Error, µg/mL
0	0	–	0	–	0	–	0	-
1	1	–	0	–	0.78	0.007	0.21	0.007
2	1.24	0.019	0.75	0.019	1.32	0.043	0.68	0.043
4	2.2	0.022	1.80	0.022	1.59	0.032	2.41	0.032
6	2.22	0.081	3.78	0.081	0.88	0.222	5.12	0.222
8	2.02	0.047	5.98	0.047	–	–	–	–

Table S3. Calculated concentrations of CRP bound to conjugates of GNP2 with different contents of IgG.

	GNP2 conjugate, 1/4 of IgG monolayer		GNP2 conjugate, 1/2 of IgG monolayer		GNP2 conjugate, IgG monolayer	
Added CRP, µg/mL	Bound CRP, µg/mL	Error, µg/mL	Bound CRP, µg/mL	Error, µg/mL	Bound CRP, µg/mL	Error, µg/mL
0	0	0	0	0	0	0
1	0.85	0.069	0.96	0.112	0.77	0.146
2	1.85	0.093	1.70	0.114	1.54	0.126
4	2.37	0.005	3.52	0.081	3.42	0.186
6	1.55	0.027	4.36	0.003	5.22	0.054
8	2.23	0.176	4.18	0.217	–	–
10	1.57	0.003	4.42	0.168	6.29	0.017
